# A novel FFQ for Brazilian adults based on the Nova classification system: development, reproducibility and validation

**DOI:** 10.1017/S1368980025000412

**Published:** 2025-03-27

**Authors:** Evelyn Oliveira da Silva Frade, Kamila Tiemann Gabe, Caroline dos Santos Costa, Daniela Neri, Euridice Martínez-Steele, Fernanda Rauber, Josiane Steluti, Renata Bertazzi Levy, Maria Laura da Costa Louzada

**Affiliations:** 1 Postgraduate Program in Nutrition and Health, Department of Nutrition, School of Public Health, University of São Paulo (USP), São Paulo, Brazil; 2 Centre for Epidemiological Studies in Nutrition and Health, School of Public Health, University of São Paulo, São Paulo, Brazil; 3 Department of Preventive Medicine, School of Medicine, University of Sao Paulo, Sao Paulo, Brazil; 4 Public Policies and Collective Health Department, Health and Society Institute, Federal University of Sao Paulo, Santos, Brazil; 5 Department of Nutrition, School of Public Health, University of Sao Paulo, Sao Paulo, Brazil

**Keywords:** Dietary intake, Nova system, Surveys and questionnaires, Nutritional epidemiology

## Abstract

**Objective:**

To describe the development and validation of the Nova FFQ (NovaFFQ) for Brazilian adults.

**Design:**

The NovaFFQ is a self-administered, semi-quantitative questionnaire. The food list includes the most consumed foods and drinks based on 2017–2018 National Food Intake Survey data. We identified and differentiated foods that could be classified into multiple Nova groups. We assessed reproducibility and criterion validity using the percent energy contribution of each Nova group. Reproducibility was assessed by comparing NovaFFQ estimates on two occasions. Criterion validity was assessed by comparing the first NovaFFQ estimate against the mean of two Nova24h recalls. We estimated the intraclass correlation coefficients (ICC) for both analyses and assessed the agreement of classification into quintiles using the prevalence-and-bias-adjusted kappa coefficients for criterion validity analysis.

**Setting:**

Nationwide Brazilian study, the NutriNet-Brasil cohort.

**Participants:**

There were 243 participants in the reproducibility analysis and 376 in the criterion validity analysis.

**Results:**

Strong reproducibility was observed, with an ICC of 0·91 for all the Nova groups. Criterion validity showed a moderate ICC, ranging from 0·61 for processed and ultra-processed foods (UPF) to 0·65 for unprocessed and minimally processed foods. Substantial agreement in ranking individuals across quintiles was found, as indicated by the prevalence-and-bias-adjusted kappa (PABAK = 0·74, 0·72, 0·70 and 0·73 for unprocessed and minimally processed foods, culinary ingredients and processed and ultra-processed foods, respectively).

**Conclusions:**

The NovaFFQ is a valid instrument for assessing food consumption by processing level, especially for discriminating individuals according to the magnitude of consumption in all Nova groups.

The Nova classification system classifies foods on the basis of the degree and purpose of industrial processing. All foods are divided into four distinct groups: (1) unprocessed or minimally processed foods, which include natural foods with minimal processing, such as cutting or grinding (e.g. fruits, vegetables, meat); (2) processed culinary ingredients, ingredients used for seasoning and cooking (e.g. sugar, salt, oil); (3) processed foods, where ingredients such as salt and sugar are added by the food industry through methods such as canning (e.g. jam, cheese) and (4) ultra-processed foods (UPF), industrial formulations made from food substances and food additives and with little or no whole foods (e.g. crackers, soft drinks, ready-to-heat or ready-to-eat meals)^(1)^.

Nova has been widely used to study the impacts of UPF on dietary patterns, health and food systems worldwide. Studies have shown, for example, a decline in the sales and consumption of unprocessed and minimally processed foods and processed culinary ingredients over time and an increase in the consumption of UPF globally^(2)^. Several of studies have documented the effects of UPF on human health. High consumption of UPF has been associated with a worse dietary nutritional profile in several countries^(3,4)^ and a greater risk of weight gain and several noncommunicable chronic diseases, such as type 2 diabetes, hypertension and some cancers^(5–7)^.

Despite this evidence, a recurring concern in the studies mentioned above is the challenging process of classifying foods according to the Nova system. As highlighted by Touvier and colleagues and Martinez-Steele and colleagues, a key limitation contributing to this challenge is the use of instruments that were not specifically developed and validated for estimating food intake within this framework. Current dietary assessment tools do not probe respondents for the level of detail necessary for researchers to make accurate Nova classifications. These studies suggest the development of new instruments specifically designed to capture these necessary details is essential for improving this issue^(8,9)^.

Traditional 24-hour recalls, which provide food-level information, often lack needed detail (e.g. whether foods are prepared at home using conventional cooking methods *v*. preprepared/packaged or brand names). To overcome the limitation of 24-hour recall, researchers from the Centre for Epidemiological Studies in Nutrition and Health at the University of São Paulo (NUPENS/USP) developed a 24-hour food recall specifically designed to assess food consumption according to the Nova system. The Nova24h is a web-based self-completed instrument that assesses foods and drinks consumed over the last 24 h. It showed good performance compared to a traditional 24-hour recall applied by an interviewer to capture the energy contribution of each Nova group and classify individuals according to quintiles of consumption of each Nova food group^(10)^.

FFQ are other instruments widely used for dietary assessment in epidemiological studies. These instruments are more easily administered than 24-hour recalls; they capture intake over a long period of time and may better estimate usual dietary intake with a single application^(11,12)^. Large prospective studies have used FFQ to assess the long-term health effects of food processing^(13–15)^. However, food misclassification is a particular concern for FFQ. Their closed food list may not include all necessary details to classify the items into Nova groups, and they may also mix home-prepared and UPF with the same item. For example, studies using these instruments may misclassify packaged cake as a culinary preparation made from unprocessed and minimally processed foods and processed culinary ingredients instead of as an ultra-processed cake^(8,9)^.

To the best of our knowledge, only three FFQ have been previously designed for estimating food consumption according to the Nova classification^(16–18)^. Motta and colleagues developed an FFQ for Brazilian children from the Midwest Region, and Amorim, Prado and Guimarães developed an FFQ for Brazilian adults from the Northeast Region. However, none of these instruments have yet been validated. Conversely, Dinu and colleagues adapted and validated an FFQ for Italian adults, which demonstrated good test-retest reliability and moderate to good validity.

Given that no instruments have been explicitly designed and validated to assess the intake of Nova groups across the Brazilian adult population, this study proposed the Nova FFQ (NovaFFQ), which is tailored for this purpose. We aimed to describe its development and evaluate the reproducibility and validity of the NovaFFQ, assessing the percent energy contribution of each Nova group among Brazilian adults.

## Materials and methods

### Development of NovaFFQ

**Figure 1.**
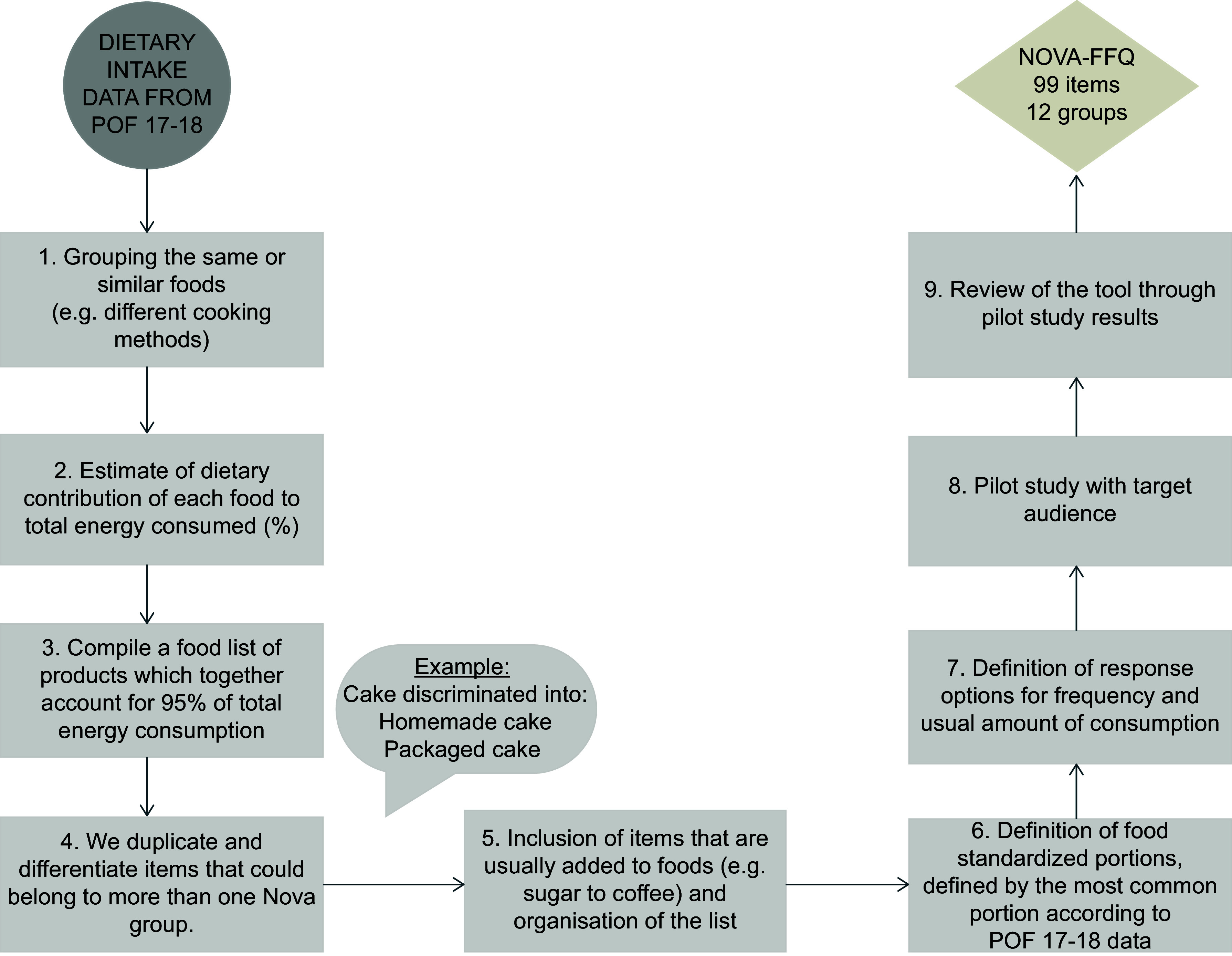
Flowchart of the development and pilot study of the NovaFFQ.

The NovaFFQ is a web-based, self-completed and semi-quantitative instrument designed to evaluate food consumption over the past twelve months. We developed the NovaFFQ in nine steps using data from 24-hour recalls of adults from the 2017 to 2018 National Food Intake Survey (POF 2017–2018). The development of the instrument is summarised in Figure 1 and detailed below.

1. We grouped identical foods that were coded differently in the database (e.g. ‘mandioca’ and ‘aipim’ are different names and codes for cassava) or that were prepared in various ways (e.g. roast meat or grilled meat).
2. The quantity of each food was converted into grams using a Brazilian reference table for foods consumed in Brazil^(19)^. These values were then transformed into kilocalories of energy using the Brazilian Food Composition Table 7.0 (TBCA)^(20)^, and the percentage of energy each food contributed to the total intake was calculated.
3. We included in a food list all foods accounting for 95 % of the calories consumed by Brazilian adults.
4. From the compiled food list, since the data from the POF 2017–2018 were collected using non-specific instruments for the Nova system, we needed to identify and differentiate each food item. To achieve this goal, two researchers with expertise in the Nova system identified all foods that could be classified into multiple Nova groups, such as yoghurt or cakes. Each of these items was replaced with two or three separate food items from the multiple Nova groups. The researchers created a description for each item, providing all relevant information for accurate identification and Nova classification. For example, for yoghurt, two different items were created: (1) ‘Flavored yogurt or ready-to-drink chocolate milk,’ classified as UPF and (2) ‘Fresh or pasteurized plain yogurt,’ classified as unprocessed and minimally processed food. Similarly, a cake was described as (1) a ‘homemade or bakery cake,’ which consists of a culinary preparation containing unprocessed and minimally processed foods and culinary ingredients or (2) a ‘store-bought, pre-packed, branded cake or prepared from a packaged mix,’ classified as ultra-processed. Online supplementary material, Supplemental material 1 includes all the items in the final NovaFFQ and its categorisation into the Nova system.
5. We included items that are usually added to foods at the time of consumption, such as sugar, butter and sauces. A team of five experts from the NUPENS/USP with experience in analysing food consumption according to Nova were invited to review the instrument. In this step, the experts analysed the suitability of the food list and the description of food items according to the Nova system.
6. For each item, we established the standardised portion as the most frequently reported portion size and unit of measure (e.g. for rice, the standardised portion was ‘1 serving spoon’) on the basis of data from the POF 2017–2018. Then, a new round of expert review was conducted to assess the definition of the standardised portions.
7. We defined the response options for the frequency of consumption and the usual amount consumed on the basis of those used by a previously validated FFQ designed to estimate the consumption of Nova food groups in Italian adults^(18)^. The options for frequency were ‘Never or rarely’, ‘1 day per month’, ‘2–3 days per month’, ‘1 day a week’, ‘2 days a week’, ‘3 days a week’, ‘4 days a week’, ‘5 days a week’, ‘6 d a week’ and ‘Daily’. The options for the usual amount consumed were presented as multiples of the standardised portion: ‘0·5’, ‘1·0’, ‘1·5’, ‘2·0’, ‘2·5’, ‘3·0’ and ‘+3·5’. Another round of expert review was conducted to assess the definitions of response options and the adjustments made to the instrument.

#### Pilot study

Next, we conducted a pilot study to verify the feasibility and interpretability of the NovaFFQ in a convenience sample of twenty adults aged eighteen years or older of both sexes residing in Brazil (depicted in steps 8 and 9 in Figure 1). We excluded pregnant or lactating women, nutrition undergraduate students and dietitians.

We recruited participants through social networks with posts on the NutriNet-Brasil study and the NUPENS account on Instagram and Twitter. We had seventy-nine applications, from which we selected twenty participants, aiming for the greatest possible diversity in terms of sex, macroregions of residence, age and schooling.

After the selected participants had filled out the consent form and NovaFFQ, we conducted an online interview to capture participants’ understanding of the initial instructions, response options for frequency and portions, standardised portions and descriptions of food items, especially with respect to food processing. We tabulated the data from each interview, and two researchers analysed and discussed the data. The average time to complete the NovaFFQ during the pilot study was twenty-five minutes. Adjustments were made to the questionnaire, followed by a final review by the five experts to assess the improvements suggested by the pilot study (step 9). The final version of the NovaFFQ was established.

### Reproducibility analysis and criterion validation

#### Study participants and data collection

We conducted NovaFFQ validation in a subsample of the ongoing NutriNet-Brasil study launched in January 2020. The NutriNet-Brasil study aimed to prospectively investigate the relationships between dietary patterns and morbidity and mortality from noncommunicable diseases in Brazil. The cohort included individuals aged eighteen years or older, with internet access, and residing in Brazil.

Every six months, participants in the NutriNet-Brasil study responded to the Nova24h recall, which was specifically developed and validated to estimate food consumption on the basis of industrial processing^(10)^.

Nova24h recall is a self-reported and web-based 24-hour recall. The participants are asked fifty-seven key questions, and then, when they answer ‘yes’ to one of them, they are presented with additional questions about the type of food (e.g. ‘homemade bread’), amount consumed (e.g. ‘1 slice’) and other details (e.g. ‘whole grain bread’). All these consisted of 395 close-ended questions. The categorisation of food items into the Nova system was conducted in a three-stage process. In the first step, two researchers independently assigned items to one of four Nova groups. Next, the classifications were reviewed by two additional researchers, and items with consensus were directly categorised. Disagreements were flagged for further review by an expert panel of researchers who created the Nova classification to reach a final consensus on the classification. Further details about Nova24h can be found in Neri *et al.* (2023)^(10)^.

The Nova24h system provides a database with all the foods and drinks consumed, including their quantities and nutritional composition, as well as the Nova classification for each item. Nutritional composition was derived by converting portions into grams and then calculating energy using data from TBCA 7.0. Within this dataset, mixed dishes are broken down into their individual ingredients using a TBCA recipe database. Nova24h was used as the reference instrument in the current study. To validate the dietary intake estimated by NovaFFQ, data from two Nova24h recalls were considered^(20)^.

We estimated a sample size of 210 participants to achieve 90 % power in detecting weak agreements (correlation coefficient = 0·2) between two observations per participant while ensuring that at least fifty individuals are included in each socio-demographic group, as recommended by another author^(11,21)^. Considering each socio-demographic category independently – sex (male and female), age (< 40 years and > 40 years), educational level (less than and more than college/university), and the five macroregions of Brazil (North, Northeast, Central-West, Southeast and South) – we defined a target total sample size of 300 individuals for reproducibility and validation.

Additionally, considering the observed refusal to respond to additional questionnaires and withdrawals from the NutriNet-Brasil study, as well as possible energy outlier reports on Nova24h, we invited 1200 participants who had completed two Nova24h recalls within the past twelve months. The NutriNet-Brasil database provides socio-demographic characteristics, including age, sex, region of residence and education level. The selection of invitees was distributed in quotas according to these variables, accounting for population distribution and ensuring a minimum of fifty participants per group. The exclusion criteria were pregnant or breastfeeding women and/or nutrition undergraduate students and dieticians.

The NovaFFQ was administered online using Google Forms. The participants accessed the questionnaire through a secure link provided via email. Only one researcher had access to the original dataset to match the NutriNet database and anonymise the responses. After the match, the original dataset was securely stored, and only the anonymised dataset was used for data analysis to ensure confidentiality and security. The participants were informed about the study procedures and completed the informed consent form. Then, they were asked to complete the NovaFFQ on two different occasions over a period of four to six weeks between administrations.

#### Data processing

The respective portions of each food reported in NovaFFQ were converted into grams and, thereafter, into energy using TBCA 7.0. The mixed dishes were disaggregated into their ingredients (e.g. home-prepared beans were broken down into beans, oil, garlic and salt) using standardised recipes from TBCA 7.0. The same criteria previously developed and validated to classify Nova24h food items according to Nova^(10)^ were applied to the NovaFFQ.

The estimated daily energy consumed from each food reported in NovaFFQ was estimated via the following equation: 
(1)

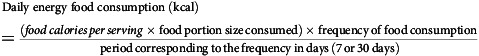




NovaFFQ items that were reported in a grouped form (e.g. rice, including white rice and brown rice) had their energy weighted for each food according to the proportion of consumption of the Brazilian population.

### Statistical analysis

We described sample characteristics with means and standard deviations for age and frequency distributions for sex (male, female), region of residence (North, Northeast, Centre-West, Southeast, South) and level of education (less than elementary, elementary, secondary, completed college/university). To compare the instruments, we estimated the percent energy contribution from each Nova group. For the Nova24h recall, we estimated the mean percent energy contribution of the two measurements.

Outliers for total energy intake estimated by NovaFFQ or Nova24h were excluded from the analysis according to the following criteria: for males, energy intake below 800 kilocalories (kcal) and above 4000 kcal; for females, energy intake below 500 kcal and above 3500 kcal^(12)^.

To evaluate the reproducibility of the instrument, the test-retest method was used. The reproducibility study sample consisted of participants who had two valid NovaFFQ assessments. We compared the percent energy contribution to the total energy intake of Nova’s groups in the first and second applications of NovaFFQ. We estimated the intraclass correlation coefficient (ICC) and 95 % CI using the two-way mixed effects model. In the reproducibility analysis, the ICC measures the degree of agreement between the individuals’ measurements taken at separate times. Values lower than 0·5 indicate poor agreement, values between 0·5 and 0·75 indicate moderate agreement, values between 0·75 and 0·90 indicate good agreement and values above 0·90 indicate excellent agreement^(22)^.

To assess the criterion validity, we compared the energy contribution of Nova’s groups obtained in the first application of the NovaFFQ against the mean estimates obtained in the two Nova24h recalls. The validation study sample was composed of participants who completed the first valid NovaFFQ assessment and two valid Nova24h assessments. We estimated the ICC and 95 % CI using a two-way mixed effects model to assess the degree of agreement between the methods. As in the reproducibility analysis, the coefficient measures the degree of agreement between the individuals’ measurements made by different instruments.

We divided the sample into quintiles of the energy contribution of each Nova group using both methods (Nova24h and NovaFFQ) to assess the ability of NovaFFQ to rank individuals according to the level of consumption of each Nova group. We estimated the proportion of participants who were correctly classified (same quintile), correctly or adjacently classified (same or next quintile) or grossly misclassified (highest quintile by NovaFFQ and lowest by Nova24h or vice versa). We also estimated the prevalence-adjusted and bias-adjusted kappa (PABAK) to assess the agreement of sample classification into quintiles. For PABAK, values between 0·00 and 0·20 indicate low agreement, values between 0·21 and 0·40 indicate acceptable agreement, values between 0·41 and 0·60 indicate moderate agreement, values between 0·61 and 0·80 indicate substantial agreement and values above 0·8 indicate almost perfect agreement^(23)^.

Bland-Altman plots were constructed to explore the agreement between the Nova24h recall and NovaFFQ and to assess the presence of systematic bias. The differences between the two methods were calculated for each participant, and these differences were plotted against the mean of the two measurements. The limits of agreement were defined as the range within which 95 % of the differences are expected to fall^(24)^.

Analyses were performed using Stata version 17.0 and R Studio software.

## Results

### Development of NovaFFQ

The initial list of foods obtained from the POF data in step 3 contained sixty-two items. After incorporating details to ensure accurate classification into the Nova groups, the number of items in the initial version of the questionnaire corresponded to 111 (step 5).

In the pilot study, some modifications were made to the tool, mainly to the section names, the descriptions of the items, the examples and the groupings of similar items. For example, the juice item initially described as ‘Natural fruit juice (fresh or pasteurized)’ was simplified to ‘Natural fruit juice’ after the pilot study because the term ‘pasteurized’ was not clear to respondents.

After adjustments, the final number of items on the NovaFFQ was ninety-nine across the twelve sections in the following order: ‘1. Cereals and pasta’; ‘2. Beans’; ‘3. Hamburgers, meats and eggs’; ‘4. Vegetables’; ‘5. Roots and tubers’; ‘6. Fruits’; ‘7. Cakes, pastries, desserts and breakfast cereals’; ‘8. Breads, biscuits, snacks and pizzas’; ‘9. Processed meat and cheese’; ‘10. Drinks’; ‘11. Nuts’ and ‘12. Items added to foods or preparations’. The respondents are provided with brief initial instructions on how to complete the NovaFFQ, and each food item included in the questionnaire has two questions: (a) frequency of consumption and (b) usual amount consumed on the basis of the standardised portion. Online supplementary material, Supplemental material 2 provides the NovaFFQ in English (which was translated freely by the authors).

### Study participants

**Figure 2.**
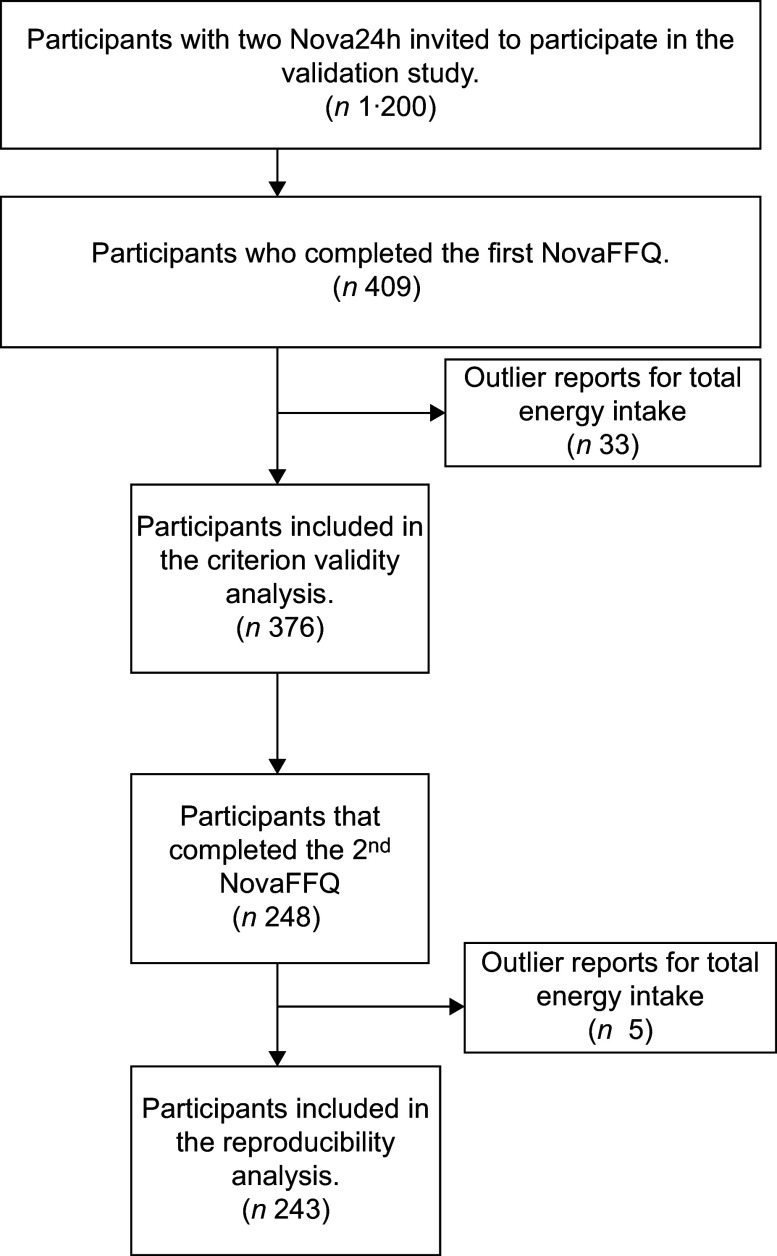
Flowchart of criterion validity and reproducibility analysis samples.

A total of 409 participants completed the first NovaFFQ. After excluding thirty-three individuals due to outlier reports for total energy intake in the Nova24h recalls and the first NovaFFQ, we had a final sample of 376 participants for validity analysis. Among the 376 participants, 248 completed the second NovaFFQ. Five participants were excluded because of outlier reports for total energy intake, resulting in a sample size of 243 for the reproducibility analysis (Figure 2).

Table 1 summarises the socio-demographic characteristics of the reproducibility and criterion validation samples. In the reproducibility sample, the participants had a mean age of 45·6 years (sd = 12·3), 55·6 % were female, 33·8 % lived in the Southeast Region and 77·4 % had completed college/university. In the criterion validation sample, the mean age was 44·2 years (sd = 12·6), 55·3 % were female, 31·4 % resided in the Southeast Region and 73·1 % had completed college/university.

**Table 1.** Socio-demographic characteristics of the study participants

Characteristics	Reproducibility sample (*n* 243)		Criterion validation sample (*n* 376)	
	*n*	%	*n*	%
Age (years)				
Mean	45·6		44·2	
sd	12·3		12·6	
Sex				
Female	135	55·6	208	55·3
Male	108	44·4	168	44·7
Region				
North	28	11·5	47	12·5
Northeast	50	20·6	79	21·0
Centre-West	46	18·9	69	18·4
Southeast	82	33·8	118	31·4
South	37	15·2	63	16·8
Educational level				
Less than elementary	9	3·7	14	3·7
Elementary	6	2·5	12	3·2
Secondary	40	16·5	75	19·9
Completed college/university	188	77·4	275	73·1

### Reproducibility analysis

The average time between the first and second measurements of the NovaFFQ was 35·4 days (sd = 2·0). The percent energy contribution of the Nova groups was similar between the NovaFFQ administrations. Unprocessed and minimally processed food presented a mean absolute difference of 0·40 percentage points (pp) (95 % CI: –0·42, 0·21), the processed culinary ingredients presented a difference of 0·10 pp (95 % CI: –0·27, 0·47), the processed foods presented a difference of 0·11 pp (95 % CI: –0·54, 0·76) and the ultra-processed group presented a difference of –0·61 pp (95 % CI: –1·28, 0·06). Additionally, we observed excellent agreement, with an ICC of 0·91 for all the Nova groups, indicating that the NovaFFQ demonstrated a good ability to produce consistent results over time (Table 2).

**Table 2.** Percent energy contribution of nova groups using the NovaFFQ applied on two different occasions. Reproducibility study (*n* 243)

Nova groups	Percent energy contribution				Mean difference^*^		ICC^†^	
	NovaFFQ 1		NovaFFQ 2					
	Mean	sd	Mean	sd		95 % CI		95 % CI
Unprocessed and minimally processed foods	55·7	11·3	55·3	11·1	0·4	−0·4, 1·2	0·91	0·88, 0·93
Processed culinary ingredients	11·7	5·1	11·6	5·0	0·1	−0·3, 0·5	0·91	0·88, 0·93
Processed foods	17·3	9·1	17·2	8·9	0·1	−0·5, 0·8	0·91	0·89, 0·93
Ultra-processed foods	15·3	9·0	15·9	9·2	−0·6	−1·3, 0·1	0·91	0·88, 0·93

* Absolute difference between the first and second administrations.

† Intraclass correlation coefficients.

### Criterion validation analysis

The average time between the first and second administrations of Nova24h was 170·2 days (sd = 22·6), whereas the average time between the second Nova24h and the first NovaFFQ was 181·8 days (sd = 43·2). The comparison of the percent energy contribution for unprocessed and minimally processed foods revealed a mean absolute difference of 5·96 pp (95 % CI: 4·70, 7·22) between the estimate of the NovaFFQ and the reference instrument (the mean of two Nova24h recalls). For processed culinary ingredients, the difference was 0·34 pp (95 % CI: –0·21, 0·89), whereas for processed and UPF, it was –1·88 pp (95 % CI: –3·01, –0·75) and –4·42 pp (95 % CI: 5·50, –3·35), respectively. We observed moderate agreement between the instruments, as indicated by the ICC ranging from 0·61 for processed and UPF to 0·65 for unprocessed and minimally processed foods (Table 3).

**Table 3.** Percent energy contribution of the nova group according to the mean of two Nova24h questionnaires and the first NovaFFQ. Criterion validation study. (*n* 376)

Nova groups	Percent energy contribution				Mean difference^*^		ICC^†^	
	Nova24h		NovaFFQ					
	Mean	sd	Mean	sd		95 % CI		95 % CI
Unprocessed and minimally processed foods	50·4	14·1	56·2	11·6	6·0	4·7, 7·2	0·65	0·48, 0·76
Processed culinary ingredients	11·3	5·5	11·7	4·9	0·3	−0·2, 0·9	0·63	0·54, 0·70
Processed foods	18·5	12·1	16·7	8·9	−1·9	−3·0, –0·8	0·61	0·52, 0·68
Ultra-processed foods	19·8	11·5	15·4	9·2	−4·4	−5·5, –3·4	0·61	0·47, 0·71

* Absolute difference between the first NovaFFQ and Nova24h.

† Intraclass correlation coefficient.

Table 4 presents the distribution of the sample into quintiles of percent energy contribution of each Nova group estimated by NovaFFQ and the reference instrument, with the cross-classification, percentage of agreement in each quintile and the PABAK statistic. Overall, we observed percentages higher than 67 % of correct or adjacent classifications and percentages lower than 15 % of gross misclassification for all the Nova groups. We also observed a greater percentage of agreement in the lowest quintile of consumption (Q1) and the highest quintile of consumption (Q5). The PABAK estimates ranged between 0·70 and 0·74, indicating substantial agreement between the instruments in ranking individuals into quintiles.

**Table 4.** Agreement and cross-classification between participant classification according to quintiles of the percent energy contribution of each Nova group estimated by the mean of two Nova24h and the first NovaFFQ (*n* 376)

Quintiles (Q) estimated by Nova24h	Quintiles (Q) estimated by NovaFFQ					Correctly classified^*^	Correctly or adjacently classified^†^	Grossly misclassified^‡^	PABAK		
	Q1	Q2	Q3	Q4	Q5					95 % CI	
Unprocessed and minimally processed foods						32·4	68·1	12·8	0·74	0·60, 0·87	
Q1	9·0	5·3	3·5	1·1	1·1						
Q2	4·3	5·1	4·5	4·5	1·6						
Q3	4·0	3·5	4·0	5·1	3·5						
Q4	2·1	5·1	4·0	4·5	4·3						
Q5	0·5	1·1	4·0	4·8	9·8						
Processed culinary ingredients						26·6	68·3	12·8	0·72	0·58, 0·86	
Q1	7·2	5·9	4·8	1·9	0·3						
Q2	5·9	3·7	5·1	3·5	1·9						
Q3	3·2	5·9	2·7	4·5	3·7						
Q4	3·2	2·7	3·2	4·8	6·1						
Q5	0·5	1·9	4·3	5·3	8·2						
Processed foods						33·0	66·7	14·6	0·70	0·55, 0·84	
Q1	7·7	4·5	3·7	2·9	1·1						
Q2	5·1	6·9	3·7	2·1	2·1						
Q3	3·7	2·7	4·5	4·8	4·3						
Q4	2·7	3·2	4·0	5·6	4·5						
Q5	0·8	2·7	4·0	4·5	8·2						
Ultra-processed foods						32·2	71·3	12·2	0·73	0·59, 0·87	
Q1	10·4	4·8	2·7	0·8	1·3						
Q2	3·2	4·5	6·1	4·8	1·3						
Q3	2·1	4·8	5·3	4·5	3·2						
Q4	3·5	4·3	3·5	3·2	5·6						
Q5	0·8	1·6	2·4	6·7	8·8						

* Correctly classified: percentage of participants classified in the same quintile.

† Correctly or adjacently classified: percentage of participants classified in the same or adjacent quintile.

‡ Grossly misclassified: percentage of participants classified in the highest quintile by first NovaFFQ and in the lowest quintile by Nova24h or vice versa.

Online supplementary material, Supplemental material 3 presents the mean percent energy contribution of the Nova subgroups estimated by the reference instrument and the NovaFFQ, as well as the difference between these estimates and the ICC of each subgroup. The largest difference between the instruments was observed for the unprocessed and minimally processed food groups, with fruits accounting for the majority of this difference (mean difference of 2·9 pp).

**Figure 3.**
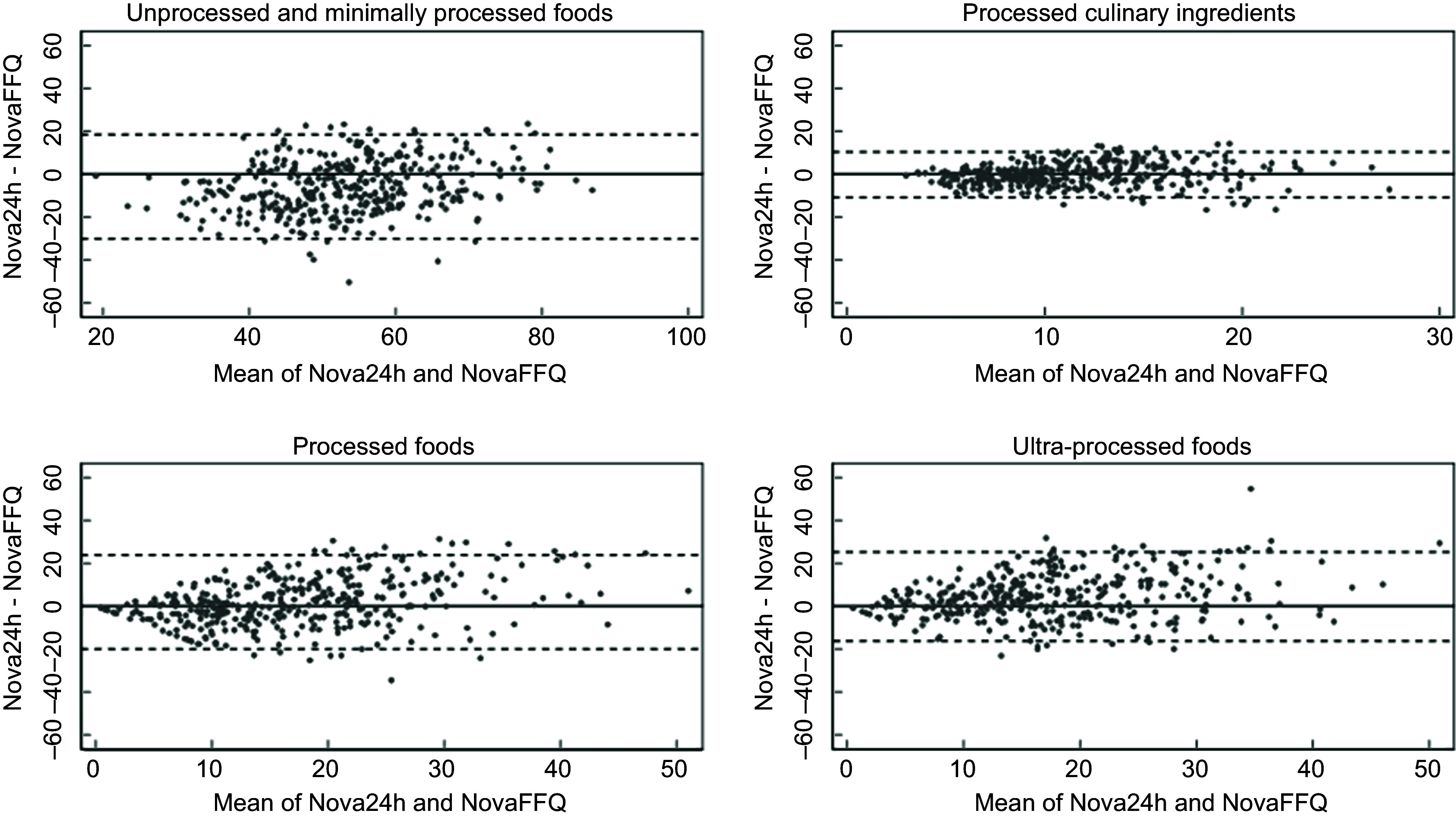
Bland-Altman plots of percent energy contribution for Nova groups estimated by the mean of two Nova24h and the first Nova FFQ.

Figure 3 presents the Bland–Altman plots, with the majority of observations within the limits of agreement. No indication of bias regarding the magnitude of consumption was found, and there was evidence of consistent agreement between the instruments for all the Nova groups.

## Discussion

This study describes the development and evaluation of the reproducibility and validity of an FFQ designed to assess food consumption in the adult Brazilian population on the basis of the Nova classification. The questionnaire underwent a rigorous review by experts in Nova classification and dietary assessment and was tested in a pilot study with Brazilian adults. The results demonstrated a strong ability to replicate energy estimates from Nova groups consistently over time and moderate criterion validity to estimate food consumption according to the Nova system. The instrument also exhibited significant validity in ranking individuals according to their level of consumption into the four Nova groups.

The NovaFFQ is the third validated instrument developed to assess food consumption on the basis of the degree of processing in the Brazilian population, together with the Nova24h recall^(10)^ and the Nova24hScreener^(25)^. The NovaFFQ is a low-cost questionnaire that can be administered repeatedly over time and, like other FFQ, may be particularly valuable for epidemiological studies aiming to assess the long-latency effects of exposure (e.g. the consumption of UPF) on outcomes such as cancer^(26)^. It may also be useful for assessing food consumption before an event that might modify food consumption, such as usual dietary intake prior to pregnancy. The significant distinction of the NovaFFQ is its capacity to assess food consumption according to food processing, providing immediate estimates of usual consumption within Nova’s four food groups.

To the best of our knowledge, only three FFQ have been specifically developed to assess food consumption according to the degree of processing. Dinu and colleagues (2021) adapted a pre-existing FFQ developed for the Italian adult population by incorporating information on food processing into the instrument. This FFQ was validated by comparing the FFQ percent energy contribution of each Nova group expressed as a percentage of grams per day against the weighted seven-day dietary record mean contributions. These authors obtained good ICC ranging from 0·77 to 0·85, similar to the moderate ICC obtained in NovaFFQ^(18)^. The other two FFQ were developed but not validated to assess dietary intake according to the Nova among Brazilians from specific regions, one for adults in the Northeast Region and the other for children in the Midwest Region^(16,17)^.

The validation analysis of the NovaFFQ indicated satisfactory agreement. The differences between the means of unprocessed/minimally processed foods may be attributed mainly to the overestimation of fruit consumption in NovaFFQ compared with Nova24h. One plausible explanation for this discrepancy could be the seasonality of fruit consumption. Since some fruits are available only during specific periods of the year, respondents may provide overestimated measures in the NovaFFQ without considering that the frequency of consumption might have varied throughout the previous year.

Another explanation refers to social desirability – the tendency to align responses with social norms to avoid criticism – which may notably affect FFQ, as these rely on individuals’ perceptions of their own diets. Awareness of the health benefits of fruit could lead participants to overreport their consumption of healthy foods. Studies have shown a positive association between social desirability (measured by a validated scale) and increased reported intake of fruits and vegetables^(27,28)^.

One of the most significant findings of the present study was the substantial agreement of the NovaFFQ to rank individuals according to the level of consumption of the four Nova groups, allowing the differentiation of high and low consumers of each group. This is particularly valuable, as most prospective studies on diet and disease incidence compare disease risk across consumption categories of dietary factors. Recently, studies have increasingly categorised participants by UPF consumption levels, using the lowest consumption group as a reference. For example, a meta-analysis of twenty-three studies revealed that the highest category of UPF consumption was associated with a 25 % and 34 % increased risk of CVD and cerebrovascular diseases, respectively^(29)^.

Previous cohort studies assessing the health effects of UPF consumption assessed by the FFQ often cite the use of FFQ not specifically designed to evaluate food processing levels as limiting. For example, Hang *et al.* (2023), in the Nurses’ Health Study, Nurses’ Health Study II and Health Professionals Follow-up Study, investigated UPF consumption and the risk of colorectal cancer precursors by comparing risk across consumption quintiles^(14)^. Similarly, a cohort from the University of Navarra (SUN, from the Spanish Seguimiento Universidad de Navarra) analysed all-cause mortality by comparing mortality between quartiles of consumption of UPF, with the first quartile used as a reference^(15)^. In Brazil, the Longitudinal Study of Adult Health (ELSA-Brazil, from the Portuguese Estudo Longitudinal de Saúde do Adulto-Brasil) evaluated the consumption of UPF and the risk of overweight and obesity by comparing the risk between the first and fourth quartiles^(13)^. Addressing this limitation underscores the relevance of the currently validated NovaFFQ.

This study has limitations and strengths. The strengths of this study include the use of data from a nationally representative survey of the Brazilian population, POF 17–18, which allowed us to incorporate the foods consumed by Brazilian adults. Additionally, the estimated sample for criterion validity analysis was achieved and presented a similar distribution of sex and macroregion of residence in relation to the general Brazilian population.

Our sample’s elevated level of schooling is a characteristic of the NutriNet-Brasil study^(30)^. This may have facilitated participants’ responses, as the NovaFFQ has a high degree of cognitive demand. However, this could limit the external validity of the results, given that only half of the Brazilian population currently completes high school^(31)^. On the other hand, to minimise this issue, we invited all individuals with lower educational levels from the NutriNet Brasil study, which allowed us to reach approximately 20 % of the sample with schooling lower than completed college/university. The intended sample size of at least fifty individuals was not reached for some specific socio-demographic groups. However, overall, we achieved a sufficient sample size for both reproducibility and validation analyses.

The use of the Nova24h recall as a reference method could be considered a limitation; however, we validated the NovaFFQ against this instrument because it was specifically designed and validated to assess food consumption on the basis of the degree of food processing^(10)^. This choice also represents a significant strength, as it minimises the risk of misclassification within the Nova system, ensuring that such errors do not compromise the validation of the NovaFFQ^(8,9)^.

Another possible limitation is the inherent correlation among the indicators utilised in our analysis, which could lead to higher ICC. We highlight that the percentage of energy is the most commonly used metric in epidemiological studies concerning ultra-processed products and health^(5–7)^. Furthermore, it has been recognised and recommended as a key parameter for monitoring diet quality^(32)^.

## Conclusions

In conclusion, the NovaFFQ has emerged as a valuable instrument that can immediately provide estimates of energy contributions from the Nova food groups for the whole Brazilian adult population. It is an instrument understood by the population of interest that has excellent reproducibility and moderate to substantial criterion validity for evaluating usual food consumption based on the degree of processing. The NovaFFQ is available on an online platform (https://questnova.com.br/) for use by researchers to assess food consumption in the Brazilian population^(33)^.

## Supporting information

Frade et al. supplementary material 1 Frade et al. supplementary material

Frade et al. supplementary material 2 Frade et al. supplementary material

Frade et al. supplementary material 3 Frade et al. supplementary material

## Supplementary material

For supplementary material accompanying this paper visit https://doi.org/10.1017/S1368980025000412
