# Supplementary material for: A novel FFQ for Brazilian adults based on the Nova classification system: development, reproducibility and validation
Source: Public Health Nutr. 2025 Mar 27;28(1):e83. doi: 10.1017/S1368980025000412 (PMC12100568; doi:10.1017/S1368980025000412)
Supplement: Frade et al. supplementary material 1 — Frade et al. supplementary material [file S1368980025000412sup001.pdf]

Supplementary material 1. Categorization of all food items in the Nova Food Frequency Questionnaire into Nova system.

Nova Food Frequency Questionnaire items	Ingredients	Nova classification
**1. Cereals, pasta and pizza**		
Corn		UMPF
Canned corn		PF
Cornflour or northeastern Brazilian couscous		UMPF
Homemade popcorn		UMPF
Microwave popcorn		UPF
Rice	Rice	UMPF
	Garlic	UMPF
	Onion	UMPF
	Soy oil	PCI
	Salt	PCI
Homemade or artisanal lasagna, ravioli, cannelloni or other homemade or artisanal stuffed pasta	Pasta sheets	UMPF
	Meat	UMPF
	Cheese	PF
	Tomato sauce	UPF
	Milk	UMPF
	Wheat flour	UMPF
	Onion	UMPF
	Garlic	UMPF
	Butter	PCI
	Oil	PCI
	Salt	PCI
Ready-to-heat pasta dishes (including stuffed pasta and lasagne)		UPF
Fresh pasta or gnocchi		UMPF
Instant noodles		UPF
Ready-to-heat pizza with brand like Sadia, Perdigão, or pizza from fast-food chains such as Pizza Hut, or Domino's		UPF
Homemade or artisanal pizza	Pizza dough	UMPF
	Cheese	PF
	Garlic	UMPF
	Onion	UMPF
	Tomato	UMPF
	Olive oil	PCI
	Salt	PCI
**2. Beans**		
Beans	Beans	UMPF
	Garlic	UMPF
	Onion	UMPF
	Oil	PCI
	Salt	PCI
Fresh pea, chickpea, or lentil	Pea, chickpea or lentil	UMPF
	Garlic	UMPF
	Onion	UMPF

	Oil	PCI
	Salt	PCI
Canned pea, or chickpea		PF
**3. Burguers, meat and egs**		
Hamburger, cheeseburger, hot dog or other fast-food snack		UPF
Steak burger, chicken or fish nuggets or sticks		UPF
Sausage, pepperoni, or frankfurter		UPF
Dried beef or jerky		UPF
Fresh beef	Beef	UMPF
	Garlic	UMPF
	Onion	UMPF
	Oil	PCI
	Salt	PCI
Fresh pork	Pork	UMPF
	Garlic	UMPF
	Onion	UMPF
	Oil	PCI
	Salt	PCI
Fresh chicken or other poultry	Chicken	UMPF
	Garlic	UMPF
	Onion	UMPF
	Oil	PCI
	Salt	PCI
Liver, tongue, gizzard, or other offal	Offal	UMPF
	Garlic	UMPF
	Onion	UMPF
	Oil	PCI
	Salt	PCI
Egg	Egg	UMPF
	Oil	PCI
	Salt	PCI
Tuna, sardines, or other fish in tin		PF
Cod or other salty fish	Cod or other salty fish	PF
	Garlic	UMPF
	Onion	UMPF
	Oil	PCI
Tilapia, hake, or other fresh fish	Fish	UMPF
	Oil	PCI
	Salt	PCI
**4. Vegetables**		UMPF
Lettuce, chard, cress or rocket		UMPF
Cabbage, kale, or spinach	Cabbage, kale, or spinach	UMPF
	Garlic	UMPF
	Onion	UMPF
	Oil	PCI
	Salt	PCI
Endive, chicory, or escarole		UMPF
Tomato or onion		UMPF

Pumpkin, zucchini, or eggplant	Pumpkin, zucchini, or eggplant	UMPF
	Salt	PCI
Carrot or beet	Carrot or beet	UMPF
	Salt	PCI
Broccoli or cauliflower	Broccoli or cauliflower	UMPF
	Salt	PCI
Chayote, okra, or green beans	Chayote, okra, or green beans	UMPF
	Salt	PCI
Other vegetables (cucumber, etc)		UMPF
**5. Roots and tubers**		
Cassava	Cassava	UMPF
	Salt	PCI
Sweet potato	Sweet potato	UMPF
	Salt	PCI
French fries frozen or purchased in fast- food stores		UPF
Potato	Potato	UMPF
	Salt	PCI
Cassava flour, homemade “farofa”	Cassava flour	UMPF
	Garlic	UMPF
	Onion	UMPF
	Oil	PCI
	Salt	PCI
Ready-to-eat “farofa”		UPF
**6. Fruits**		
Banana		UMPF
Orange, or tangerine		UMPF
Apple or pear		UMPF
Papaya		UMPF
Mango		UMPF
Watermelon or melon		UMPF
Others fruits (grape, pineapple, etc)		UMPF
**7. Cakes, candy, desserts and breakfast cereals**		
Fruit in syrup, or candy made from fruit (e.g. cocada, goiabada)		PF
Candy made from peanut (paçoca, pé-de-moleque)		PF
Milk caramel, or hazelnut butter		UPF
Homemade flan, coconut pudding, mousse, rice pudding, trifle made from the ingredients	Eggs	UMPF
	Milk	UMPF
	Corn starch	PCI
	Sugar	PCI
	Condensed milk	UPF
Ready-to-eat or powdered flan, coconut pudding, mousse, rice pudding, or trifle		UPF
Porridge, hominy porridge, or corn pudding.	Milk	UMPF
	Sugar	PCI
	Corn starch	PCI
Gelatin		UPF
Chocolate, or truffle		UPF

Ice cream		UPF
Cookie with or without filling		UPF
Homemade cake	Eggs	UMPF
	Wheat flour	UMPF
	Milk	UMPF
	Butter	PCI
	Sugar	PCI
Ready-to-eat or powdered cake		UPF
Breakfast cereals or granola		UPF
**8. Breads and crackers**		
Homemade bread	Wheat flour	UMPF
	Water	UMPF
	Yeast	PCI
	Sugar	PCI
	Salt	PCI
Freshly made unpackaged bread (homemade or artisanal)		PF
Sandwich bread		UPF
Others packaged breads		UPF
Tapioca		PCI
Homemade or bakery Brazilian cheese bread, savory fried or baked, or pie	Eggs	UMPF
	Water	UMPF
	Tapioca starch	PCI
	Oil	PCI
	Salt	PCI
	Cheese	PF
Frozen or packaged Brazilian cheese bread, savory fried or baked, or pie		UPF
Crackers, packaged toast		UPF
Snacks		UPF
**9. Processed meats and cheese**		
Ham, mortadella, or salami		UPF
Mozzarella cheese, or other cheese		PF
**10. Drinks**		
Milk		UMPF
Flavoured yoghurt or ready- to-drink chocolate milk		UPF
Milk with chocolate powder	Milk	UMPF
	Chocolate powder	UPF
Fresh or pasteurized plain yoghurt		UMPF
Coffee		UMPF
Milk with coffee		UMPF
Fresh 100% fruit juice		UMPF
Soft drinks (e.g. iced tea, fruit juice) or energy drinks		UPF
Tea and herbal infusions		UMPF
Soda		UPF
Beer		PF
Wine		PF
Vodka, whiskey or others drinks alcoholic distilled		UPF
**11. Nuts**		

Packaged peanut with brand	UPF
Peanut, nuts with salt or sugar	PF
Peanut, nuts without salt or sugar	UMPF
**12. Items added to ready dishes**	
Sugar	PCI
Sweetener	UPF
Olive oil or oil	PCI
Butter	PCI
Margarine	UPF
Ketchup, mustard, mayonnaise, soy sauce or other ready-made sauces	UPF
Cottage cheese or cream cheese	UPF
Jam	PF
Packaged grated cheese	UPF
Freshly grated parmesan cheese	PF

UMPF: Unprocessed and minimally processed foods
PCI: Processed culinary ingredients
PF: Processed foods
UPF: Ultra-processed foods
