# Supplementary material for: A novel FFQ for Brazilian adults based on the Nova classification system: development, reproducibility and validation
Source: Public Health Nutr. 2025 Mar 27;28(1):e83. doi: 10.1017/S1368980025000412 (PMC12100568; doi:10.1017/S1368980025000412)
Supplement: Frade et al. supplementary material 2 — Frade et al. supplementary material [file S1368980025000412sup002.pdf]

Supplementary material 2. NovaFFQ in English (free translation).

Participant,

Here you will answer about your usual diet over the last twelve months.
For each listed item, answer by selecting:

1) your frequency of consumption (ranging from “never or <1 time per month” to "daily").
2) your typical portion size consumed using the reference portion available for each item (ranging from “0.5” to “>3,5” portions).

For example, if you usually consume two units of bread per day, select the frequency "daily" and the option "two" for the portion size, knowing that the reference portion it is "1 unit ".

For items that contain more than one food (example: melon or watermelon), record the sum of how often you eat both fruits and the portion size you usually eat.

In the final section, you must record the consumption of items such as sugar, olive oil and ketchup added to drinks, salads, or pizza.

Let's start?

How often have you eaten the following during the past 12 months? Select **only one** frequency per item.											How much did you eat each per day (considering the sum of all meals of a day)? Indicate **only one** portion per item. If the response to frequency is "Never or rarely" no need to mark answer for that question.							
Food	Never or rarely	1 day per month	2-3 days per month	1 day a week	2 days a week	3 days a week	4 days a week	5 days a week	6 days a week	Daily	Reference portion	0.5	1.0	1.5	2.0	2.5	3.0	+3.5
**1. CEREALS, PASTA, AND PIZZA**																		
Corn											1 cob							
Canned corn											1 tablespoon							
Cornflour or northeastern Brazilian couscous											1 serving spoon							
Homemade popcorn											1 bowl average full							
Microwave popcorn											1 package							
Rice											1 serving spoon							
Homemade or artisanal lasagna, ravioli, cannelloni or other homemade or artisanal stuffed pasta											1 piece or 2 servings spoons							

Ready-to-heat pasta dishes (including stuffed pasta and lasagne)											1 piece medium or 2 serving spoons							
Fresh pasta or gnocchi											1 serving spoon							
Instant noodles											1 pack							
Ready-to-heat pizza with brand like Sadia, Perdigão, or pizza from fast-food chains such as Pizza Hut, or Domino's											1 slice							
Homemade or artisanal pizza											1 slice							
**2. BEANS AND LEGUMES**																		
Beans											1 ladle							
Fresh pea, chickpea, or lentil											1 tablespoon							

Canned pea, or chickpea											1 tablespoon							
**3. BURGERS, MEAT AND EGGS**																	
Hamburger, cheeseburger, hot dog or other fast-food snack											1 unit average							
Steak burger, chicken or fish nuggets or sticks											1 unit of steak burger or 6 units of nuggets							
Sausage, pepperoni, or frankfurter											3 slices of sausage or pepperoni or 1 average frankfurter							
Dried beef or jerky											1 average piece							
Fresh beef											3 medium pieces or 1 steak or serving spoon							

Fresh pork											1 average slice or 3 ribs							
Fresh chicken or other poultry											1 medium thigh or 1 fillet or 2 medium drumsticks							
Liver, tongue, gizzard, or other offal											1 average piece							
Egg											1 unit							
Tuna, sardines, or other fish in tin											1 tablespoon							
Cod or other salty fish											1 average piece							
Tilapia, hake, or other fresh fish											1 average piece							
**4. VEGETABLES**																		
Lettuce, chard, cress or rocket											1 average leaf							

Cabbage, kale , or spinach											1 tablespoon							
Endive, Chicory, or escarole											1 ladle							
Tomato or onion											1 tomato slice or 2 onion slices							
Pumpkin, zucchini, or eggplant											1 serving spoon							
Carrot or beet											1 tablespoon							
Broccoli or cauliflower											1 average floret							
Chayote, okra, or green beans											1 tablespoon							
Other vegetables (cucumber, etc)											1 tablespoon							
**5. ROOTS AND TUBERS**																		
Cassava											1 average piece							
Sweet potato											1 average piece							

French fries frozen or purchased in fast-food stores											1 average serving or 1/2 dish							
Potato											1 small unit							
Cassava flour, homemade *"farofa"*											1 tablespoon							
Ready-to-eat *"farofa"*											1 tablespoon							
**6. FRUITS**																		
Banana											1 unit average							
Orange, or tangerine											1 unit average							
Apple or pear											1 unit average							
Papaya											1 slice average							
Mango											1 unit average							

Watermelon or melon											1 slice average of watermelon or 2 slice average of melon							
Others fruits (grape, pineapple, etc)											1 slice average							
**7.CAKES, CANDY, DESSERTS AND BREAKFAST CEREALS**																		
Fruit in syrup, or candy made from fruit (e.g. *cocada*, *goiabada*)											1 unit average or 1 tablespoon							
Candy made from peanut (*paçoca*, *pé-de-moleque*)											1 unit average							
Milk caramel, or hazelnut butter											1 tablespoon							
Homemade flan, coconut pudding, mousse, rice pudding, trifle made from the ingredients											1 piece average or 1 serving spoon							

Ready-to-eat or powdered flan, coconut pudding, mousse, rice pudding, or trifle											1 piece average or 1 serving spoon							
Porridge, hominy porridge, or corn pudding.											1 dish							
Gelatin											1 bowl average							
Chocolate, or truffle											1 unit average							
Ice cream											1 serving							

Cookie with or without filling											2 units of cookie without filling or 1 unit of cookie stuffed							
Homemade cake											1 slice average							
Ready-to-eat or powdered cake											1 unit small or 1 slice average							
Breakfast cereals or granola											1 bowl of breakfast cereal or 3 tablespoons of granola							
**8.BREADS AND CRACKERS**																		
Homemade bread											1 slice average							
Freshly made unpackaged bread (homemade or artisanal)											1 unit average or 2 slices							

Sandwich bread											1 slice average							
Others packaged breads											1 unit average							
Tapioca											1 unit average							
Homemade or bakery Brazilian cheese bread, savory fried or baked, or pie											1 unit average							
Frozen or packaged Brazilian cheese bread, savory fried or baked, or pie											1 unit average							
Crackers, packaged toast											1 unit average							
Snacks											1 pack average							
**9. PROCESSED MEATS AND CHEESE**																		

Ham, mortadella, or salami											1 slice average							
Mozzarella cheese, or other cheese											1 slice average							
**10. DRINKS**																		
Milk											1 glass							
Flavoured yoghurt or ready-to-drink chocolate milk											1 glass							
Milk with chocolate powder											1 glass							
Fresh or pasteurized plain yoghurt											1 glass							
Coffee											1 cup							
Milk with coffee											1 cup							
Fresh 100% fruit juice											1 glass							
Soft drinks (e.g. iced tea, fruit juice) or energy drinks											1 glass							

Tea and herbal infusions											1 cup							
Soda											1 glass							
Beer											1 can or 1 glass							
Wine											1 glass							
Vodka, whiskey or others drinks alcoholic distilled											1 shot							
**11. NUTS**																		
Packaged peanut with brand											1 pack small							
Peanut, nuts with salt or sugar											1 serving average							
Peanut, nuts without salt or sugar											1 serving average							
How often you added those items to ready foods in the last twelve months? (Example: sugar at the coffee, oil at salad, margarine at the bread) Select **only one** option per item.											How much did you added to foods per day (considering the sum of all meals of a day)? Indicate **only one** portion per item. If the response to frequency is "Never or rarely" no need to mark answer for that question.							
**12. ITEMS ADDED TO READY DISHES**																		
Sugar											1 teaspoon							
Sweetener											3 drops or 1 sachet							
Olive oil or oil											1 tablespoon							

Butter											1 knife tip							
Margarine											1 knife tip							
Ketchup, mustard, mayonnaise, soy sauce or other ready-made sauces											1 tablespoon							
Cottage cheese or cream cheese											1 knife tip							
Jam											1 knife tip							
Packaged grated cheese											1 tablespoon							
Freshly grated parmesan cheese											1 tablespoon							
