# Supplementary material for: A novel FFQ for Brazilian adults based on the Nova classification system: development, reproducibility and validation
Source: Public Health Nutr. 2025 Mar 27;28(1):e83. doi: 10.1017/S1368980025000412 (PMC12100568; doi:10.1017/S1368980025000412)
Supplement: Frade et al. supplementary material 3 — Frade et al. supplementary material [file S1368980025000412sup003.pdf]

Supplementary material 3. Table S1. Percent energy contribution of Nova subgroups using the Nova24h and the first Nova Food Frequency Questionnaire. Criterion validation study. (n=377)

**Nova groups and subgroups**	**Percent energy contribution**				**Mean difference[1]**			**ICC[2]**		
	**Nova24h**		**NovaFFQ**							
	**Mean**	**SD**	**Mean**	**SD**		**95% CI**			**95% CI**	
**Unprocessed or minimally processed foods**	**50.4**	**14.1**	**56.2**	**11.6**	**6.0**	**4.7**	**7.2**	**0.65**	**0.48**	**0.76**
Fruit	7.8	6.5	10.7	7.6	2.9	2.2	3.6	0.69	0.54	0.78
Red meat	7.3	8.2	8.5	7.0	1.2	0.3	2.0	0.60	0.52	0.68
Milk and plain yoghurt	3.6	4.4	4.8	5.3	1.1	0.6	1.6	0.65	0.56	0.71
Eggs	3.2	3.7	4.3	4.0	1.1	0.7	1.4	0.73	0.65	0.79
Poultry	3.3	4.5	4.0	3.4	0.7	0.2	1.2	0.39	0.26	0.50
Legumes	4.5	4.3	5.0	4.0	0.5	0.1	1.0	0.62	0.53	0.69
Pasta	2.1	4.1	2.6	2.6	0.5	0.1	1.0	0.35	0.21	0.47
Roots and tubers	1.8	2.9	2.1	2.2	0.3	0.0	0.6	0.49	0.37	0.58
Homemade pies, pastries, pizza	1.0	3.4	1.1	1.4	0.1	-0.3	0.5	0.05	-0.17	0.22
Coffee and tea	0.6	0.5	0.7	0.5	0.0	0.0	0.1	0.71	0.65	0.76
Vegetables	1.7	1.2	1.6	0.9	-0.1	-0.2	0.0	0.52	0.41	0.61
Flour	1.2	2.0	0.9	1.6	-0.2	-0.4	0.0	0.59	0.49	0.66
Grains	5.7	4.1	5.4	3.4	-0.2	-0.6	0.2	0.59	0.49	0.66
Freshly squeezed fruit juice	2.0	2.8	1.7	2.6	-0.3	-0.6	0.0	0.59	0.49	0.66
Cassava flour	1.1	2.4	0.7	1.7	-0.4	-0.6	-0.2	0.75	0.69	0.80
Nuts and seeds without salt, sugar, or oil	1.6	3.2	1.2	2.3	-0.5	-0.8	-0.2	0.55	0.45	0.64
Fish and seafood	1.6	3.4	1.0	1.5	-0.6	-0.9	-0.3	0.26	0.10	0.40

Other unprocessed or minimally processed foods	0.2	1.0	0.0	0.0	-0.2	-0.3	-0.1	0.14	-0.05	0.30
**Processed culinary ingredients**	**11.3**	**5.5**	**11.7**	**4.9**	**0.3**	**-0,2**	**0.9**	**0.63**	**0.54**	**0.70**
Plant oils	4.8	2.0	5.9	2.0	1.1	0.8	1.4	0.55	0.38	0.66
Animal fats	1.8	1.7	1.6	1.7	-0.2	-0.4	0.0	0.58	0.48	0.65
Sugar	3.1	3.2	1.9	1.9	-1.3	-1.6	-1.0	0.47	0.30	0.59
Other processed culinary ingredients	1.6	2.5	2.3	2.8	0.7	0.4	1.0	0.60	0.49	0.69
**Processed foods**	**18.5**	**12.1**	**16.7**	**8.9**	**-1.9**	**-3.0**	**-0.8**	**0.61**	**0.52**	**0.68**
Ham and other salted, smoked, or canned meat or fish	0.7	1.7	1.8	2.2	1.1	0.8	1.3	0.26	0.09	0.40
Wine and beer	3.6	6.8	4.0	5.9	0.4	-0.2	1.0	0.73	0.66	0.78
Canned vegetables and legumes	0.1	0.3	0.1	0.2	0.0	0.0	0.0	0.08	-0.13	0.25
Processed bread	3.5	4.4	3.4	4.2	0.0	-0.5	0.4	0.54	0.43	0.62
Dried and canned fruit	0.2	1.9	0.1	0.3	-0.1	-0.3	0.1	0.19	0.00	0.34
Nuts and seeds with salt or sugar	0.6	2.1	0.5	1.4	-0.1	-0.3	0.1	0.36	0.22	0.48
Processed cake	1.2	3.3	0.8	1.1	-0.4	-0.7	0.0	0.07	-0.14	0.24
Processed desserts	0.5	1.8	0.1	0.6	-0.4	-0.6	-0.2	-0.03	-0.25	0.15
Savoury snacks, including croquettes, pastries, and mini pies	1.2	3.1	0.7	1.1	-0.5	-0.8	-0.2	0.12	-0.07	0.28
Cheese	7.0	6.3	5.1	3.7	-1.9	-2.4	-1.3	0.55	0.42	0.64
**Ultra-processed foods**	**19.8**	**11.5**	**15.4**	**9.2**	**-4.4**	**-5.5**	**-3.35**	**0.61**	**0.47**	**0.71**
Breakfast cereals	0.4	1.2	0.6	1.8	0.1	0.0	0.3	0.69	0.62	0.75
Bread	2.5	3.4	2.6	3.1	0.1	-0.3	0.4	0.55	0.44	0.63
Other sweetened beverages (fruit juices, energy drinks)	0.5	2.0	0.5	1.8	0.0	-0.2	0.3	0.57	0.47	0.65
Soda	0.8	2.3	0.9	2.1	0.0	-0.2	0.2	0.62	0.53	0.69
Pre-cooked French fries	0.3	1.0	0.2	0.6	0.0	-0.1	0.1	-0.02	-0.24	0.17
Sauces, dressings, and gravies	0.6	1.0	0.5	0.8	0.0	-0.1	0.1	0.25	0.08	0.38
Instant and canned soups	0.2	1.8	0.2	0.9	0.0	-0.2	0.2	0.07	-0.14	0.24
Cream cheese	0.3	0.7	0.3	0.5	0.0	-0.1	0.0	0.55	0.45	0.63

Desserts	0.9	2.2	0.8	1.2	-0.1	-0.3	0.2	0.29	0.13	0.42
Milk-based drinks and flavoured yoghurts	0.9	2.1	0.8	1.9	-0.1	-0.3	0.1	0.55	0.44	0.63
Margarine	0.4	1.1	0.2	0.6	-0.1	-0.2	0.0	0.59	0.50	0.66
Ready-to-heat or ready-to-eat meals	0.5	2.2	0.2	0.6	-0.3	-0.5	-0.1	0.23	0.06	0.37
Reconstituted meat or fish products	2.9	4.1	2.6	2.2	-0.3	-0.7	0.1	0.44	0.31	0.54
Ready-to-heat pizza and pies	0.8	2.8	0.4	1.2	-0.4	-0.7	-0.1	0.02	-0.19	0.20
Ice cream	1.1	2.7	0.6	1.2	-0.4	-0.7	-0.2	0.28	0.12	0.41
Vodka, tequila, and other distilled alcoholic drinks	0.7	2.8	0.3	1.0	-0.5	-0.7	-0.2	0.40	0.27	0.51
Crackers	1.6	3.5	1.1	1.8	-0.5	-0.9	-0.2	0.35	0.21	0.47
Sweets	2.3	3.4	1.6	1.9	-0.7	-1.0	-0.3	0.36	0.22	0.48
Cakes, cookies, and pies	1.4	3.0	0.8	1.4	-0.7	-1.0	-0.4	0.29	0.13	0.42
Other ultra-processed foods	0.7	1.5	0.2	0.5	-0.5	-0.6	-0.3	0.19	0.01	0.34

[1] Absolute difference between the first NovaFFQ and the Nova24h

[2] Intraclass correlation coefficients
